# Over the counter: The potential for easing pharmacy provision of family planning in urban Senegal

**DOI:** 10.12688/gatesopenres.12825.3

**Published:** 2019-05-15

**Authors:** Jill Peterson, Aurelie Brunie, Ibrahima Diop, Seynabou Diop, John Stanback, Dawn S. Chin-Quee

**Affiliations:** 1 FHI 360, Washington, DC, 20009, USA; 2 Agence pour la Promotion des Activités de Population-Sénégal (APAPS), Dakar, Senegal; 3 Marie Stopes International, Dakar, Senegal; 4 FHI 360, Durham, NC, 27701, USA

**Keywords:** Pharmacy, Contraception, Family Planning, Senegal

## Abstract

**Background:** This research assessed the potential for expanding access to family planning through private sector pharmacies in Senegal, by examining the quality of the services provided through private sector pharmacies, and pharmacy staff and client interest in private sector pharmacy-based family planning services.

**Methods:** This was a cross-sectional, descriptive study conducted in eight urban districts in and around Dakar and two urban districts outside of Dakar employing an audit of 225 pharmacies, a survey with 486 private sector pharmacy staff and a survey with 3,567 women exiting private sector pharmacies.

**Results:** Most (54%) pharmacies reported offering method-specific counseling to clients. Family planning  commodities were available in all pharmacies, and 72% had a private space available to offer counseling. Three quarters (76%) did not have any counseling materials available.

49% of pharmacists and 47% of assistant pharmacists reported receiving training on family planning during their professional studies. Half had received counseling training. Few pharmacists met pre-determined criteria to be considered highly knowledgeable of the oral contraceptive pill (OCP) and injectable contraceptive provision (0.6% and 1.1%).

Overall, 60% of women surveyed were current family planning users and 11% procured their method through a private sector pharmacy. Among non-users of family planning, and current users who did not obtain their method through a pharmacy, 47% said they would be interested in procuring a method through a private sector pharmacy.

**Conclusions:** There is both actual and latent demand for accessing family planning through Senegal’s urban, private sector pharmacies. With proper training, pharmacy staff could better provide effective counseling and provision of OCPs and injectables, and lifting the requirement for a prescription could help support gains in contraceptive prevalence.

## Introduction

Limited access to family planning services has been linked to the high rates of unmet need for contraception found in many African countries ^1^. Over the years, many countries have attempted to fill gaps in health services by capitalizing on private sector facilities, including privately run clinics, social franchising, and pharmacies ^2^. Research has shown drug shops, which typically do not employ a licensed pharmacist and are normally restricted to selling only over-the-counter medications, and private sector pharmacies to be a major source for contraceptives, particularly oral contraceptive pills (OCPs), emergency contraception, and condoms, especially for hard to reach women such as those who are younger or unmarried ^3– 5^.

Hand in hand with increasing access through the private sector is task-sharing, in which lower level cadres of health providers are trained to provide certain health services that previously were the sole purview of a higher cadre ^6^. Task sharing is a mechanism used in many countries to increase access to a variety of health services, including family planning. For example, community health workers in the public sector, and pharmacy and drug shop staff in the private sector, have all been trained in various countries to provide OCPs and injectable contraception—the two most popular forms of contraception in sub-Saharan Africa—without the need for a prescription written by a higher-level clinician, even when it is the first time the woman is using the method ^7^.

In urban settings in particular, task-sharing of OCPs and injectables through private sector pharmacies is promising for improving access to family planning services. Thanks to the range of possible reasons for visiting pharmacies, the purpose of the visit can be disguised, providing a layer of confidentiality to women. In addition, research has shown that direct private sector pharmacy access to OCPs poses little risk to women; appropriately trained pharmacy staff are able to counsel for and provide the pill ^8, 9^, and further research has shown that women can even self-screen for eligibility of OCPs with relative accuracy by self-administering a checklist ^10, 11^. Sale and provision of injectable contraceptives in private sector pharmacies and drug shops is also common in the developing world, thanks to social marketing ^12, 13^. Lastly, new packaging of injectable contraception designed to allow women to self-inject could eliminate the need for administration by a skilled professional for many, further increasing the impact pharmacies could have in improving access to contraception ^14^.

At the same time, there are some possible concerns associated with over-the-counter provision of family planning services through pharmacies. Historically, low-income clients have been priced out of private facility services, although several programs are working to decrease financial barriers, including by increasing access to insurance and implementing savings products and voucher systems ^15^. Screening for underlying health conditions is always desirable, but may not occur during a pharmacy visit. Similarly, private sector pharmacies may do little to provide re-supply reminders to clients buying methods such as OCPs and injectables. And, finally, private pharmacies may not counsel and refer women for methods they do not sell, since they are not usually integrated into the larger public health system, and since there is no economic incentive for them to do so.

To date, research focused on family service provision in pharmacy settings is limited and outdated. This research focused on Senegal seeks to address this gap. Senegalese pharmacies are ubiquitous in urban areas compared to health facilities. For example, Dakar counts 490 pharmacies but only 34 health facilities. Both public and private sector pharmacists are currently forbidden from dispensing OCPs and injectable contraception without a prescription, and pharmacists are not allowed to write prescriptions themselves. However, the Senegalese National Family Planning Action Plan for 2012–2015 ^16^ acknowledged the barriers imposed by a prescription requirement as a limitation to the regulatory framework of family planning and the expansion of family planning in Senegal. The removal of prescription barriers was also noted in Senegal’s 2012 Program and Service Delivery commitment to Family Planning 2020 (FP 2020), and the updated National Strategic Framework for Family Planning 2016–2020 ^17^, calls for finalizing and validating the laws and regulations governing the pharmaceutical sector. 

As an estimated 44% of Senegal’s 14 million people live in an urban setting, and 38% of the total population living on less than $1.90 per day, urban poverty is a concern ^18, 19^. Given a total fertility rate of 5.0, a contraceptive prevalence rate (CPR) of 20.3%, and an unmet need for family planning at 25.6% overall, rising to nearly 30% for the lowest wealth quintile, increasing family planning access amongst the poorest women could help Senegal reach its commitment to FP 2020 with a CPR of 45% by 2020 ^20^. Senegal sees the need and is clearly interested in updating their national policies regarding accessing family planning through pharmacies, and this research aims to provide evidence as they work to make final decisions.

This article reports on research conducted by FHI 360 under the Bill & Melinda Gates Foundation-funded Urban Reproductive Health Initiative (UHRI), led by IntraHealth International in Senegal, to assess the potential for expanding access to family planning through pharmacies. Specific objectives were to assess the quality of the services currently provided through pharmacies, and to examine pharmacy staff and client interest in pharmacy-based family planning services. This research aimed to provide evidence for a potential policy change that would help Senegal reach its family planning goals by expanding access through pharmacies.

## Methods

This was a cross-sectional, descriptive study employing a pharmacy audit, a survey with pharmacy staff, and a survey with pharmacy clients.

### Design and selection procedures

We obtained a list of all private sector pharmacies (551) in all 10 URHI project districts,. Eight of the project districts were in Dakar and two were urban districts outside of Dakar (Mbour and Kaolack). The study primary outcome was the proportion of private sector pharmacies that provide counseling for family planning methods as measured through self-reported pharmacy audits. We assumed that 65% of the pharmacies in our target districts would have received training on family planning counseling through one of two earlier projects funded for pharmacy training (the Health Services Improvement project, or RPS in French, and the Senegal Maternal, Newborn, and Child Health/Family Planning/ Malaria project). Thus, we determined that a sample size of 225 pharmacies would be needed to achieve a 95% confidence interval with five percent precision for the proportion of interest including a finite population correction. To be conservative we planned on sampling 250 pharmacies to allow for 10% refusal. A proportional random sample of pharmacies stratified by district was selected.

Pharmacy audit: Eligibility criteria for pharmacies included location within UHRI districts and willingness of the owner of the pharmacy to participate in the study. We attempted to speak with the most knowledgeable person present, or the one who had worked there the longest, but collective input was also allowed.

Pharmacy staff: In each selected pharmacy, we attempted to interview one pharmacist, one assistant pharmacist, and one counter staff or trainee. If no personnel from any one of the three positions were present the day of the interviews, that position was not included. When more than one was present for a given position, the one whose first name came first alphabetically was selected.

Clients: Eligible clients were women (aged 18–49, plus married women aged 15–17) exiting the selected pharmacies over a two-day period for each pharmacy. Initially, we conducted exit interviews with all women of reproductive age (as assessed by the survey takers) regardless of current family planning use in the first 50 pharmacies. Subsequently, after the first 50 we made the decision to cap the number of non-users of family planning interviewed at five per pharmacy to ensure data collectors had enough time to find and interview users of family planning. We interviewed all current users of family planning who consented, regardless of whether they sourced their method through the pharmacy or elsewhere.

### Data collection

Prior to initiating the study in any pharmacy, the research staff reached out to pharmacy owners and explained the purpose of the research, emphasizing confidentiality and that there was no threat of penalty from the regulatory division of the Ministry of Health and Social Action, the *Direction de la Pharmacie et du Médicament* (DPM), for any findings or responses and that responses would not be linked to particular pharmacies. Trained female data collectors conducted pharmacy audits, interviews with pharmacy staff, and client interviews between March and June 2015. Interviews with pharmacy staff, including for the audit, were conducted inside, whereas clients were intercepted and interviewed outside of the pharmacy so that pharmacy staff would not overhear their responses. Data collectors were required to have the equivalent of a technical degree ( *Brevet de Fin d'Etudes Moyennes*) and to have previously participated in at least three household surveys, but they did not have clinical training.

Written informed consent was obtained from all participants prior to conducting each type of interview in French, Wolof, or other local languages. No compensation was provided. The study was approved by Senegal’s national ethics committee ( *Comité National d'Ethique pour la Recherche en Santé;* approval number, SEN14/25) and FHI 360’s Protection of Human Subjects Committee (564606-1).

### Analysis methods

Table 1 outlines the information gathered through each of the three questionnaires (questionnaires are available on Harvard Dataverse ^21^). Data from each survey were analyzed descriptively using SPSS version 17.0. We constructed two separate composite indicators of pharmacy staff knowledge of OCPs and injectable contraception based on responses to the survey with pharmacy staff. Points were awarded as shown in tables (see Results section). While we did not strictly adhere to a theoretical quality of care framework such as Bruce-Jain ^22^, questions were derived from tools such as the Quick Investigation of Quality ^23^, and the Situation Analysis, which are based on this framework ^24^. To simplify scoring and to avoid endless debate about the relative weights of the various questions, all questions were assumed to have equal importance. We considered staff who were able to provide correct responses to half or more of the questions (five or more out of eight points for OCPs and three or more out of six points for injectables) to be knowledgeable of each method. While this is a crude measure, we reasoned it was sufficient in the context of an exploratory study that aimed at providing initial insight into staff knowledge rather than precisely measuring competency. 

**Table 1.** Data collection methods and information gathered.

Data collection method	Information gathered
Audit/situation analysis	• Availability and conditions of family planning method provision, • Presence of support materials in-house, and • Availability of a private area (visual and/or auditory) to conduct counseling
Staff interview	• Knowledge and provision of family planning services, • Interest in providing family planning services, and • Receipt and/or desire for (additional) training in family planning service delivery
Client exit intercept interviews	• Client experiences with and demand for pharmacy-based family planning services

## Results

In total, we performed audits at 225 pharmacies, and interviewed 486 pharmacy personnel and 3,567 pharmacy clients. From the sample of 250 pharmacies drawn from our initial list, 20 refused to participate in the study and 11 were either permanently or temporarily closed, or in the process of being sold, four were outside of our study districts, and one was sampled twice. To meet our target size of 225, we randomly selected an additional 11 pharmacies (respecting the initial stratification by district). All 11 replacement pharmacies were open and consented to participate. Of the 486 pharmacy personnel interviewed, 182 were pharmacists, 94 were assistant pharmacists, 206 were counter staff, and 4 were interns. In total 56% of pharmacy staff respondents were men.

### Client demographics

Of the total 3,567 clients interviewed, 390 (11%) were current family planning users who procured their method through a private sector pharmacy, 1,676 (47%) were current users of family planning who procured their method elsewhere, and 1,436 (40%) were non-users of family planning. The remaining 2% were using a method that did not need procuring, such as lactational amenorrhea, or were users of traditional methods. The average age of our respondents was 30.6 years. Our respondents were most commonly married (76%), living with their partner (83%) and had a previous pregnancy (79%). In total, 53% had one to two children, and the most common education level was primary (29%) (see Table 2).

**Table 2.** Demographic information for clients interviewed (n=3,567).

Question	% (n)
Average age	30.6
Marital status Married Single Separated/Divorced Widowed/Other	76 (2,718) 18 (651) 5 (175) 1 (21)
Of those with partner: Live with partner Do not live with partner	83 (2,280) 17 (480)
Previous pregnancy Yes No	79 (2,807) 21 (760)
Number of living children 0 1–2 3–4 5–6 >6	2 (49) 53 (1,493) 32 (898) 10 (290) 3 (77)
Education level No formal schooling Primary Secondary 1 Secondary 2 Post-Secondary Other	22 (771) 29 (1,042) 17 (608) 12 (407) 16 (566) 5 (159)

### Audit

Overall, 54% (95% CI: 48%–61%) of pharmacies reported offering counseling on at least one method of family planning method. On the day of the audit, 99% of pharmacies had at least one brand of OCP in stock; the most common was Securil (approximately 0.70 USD), stocked by 98%. Between 73 and 79% also reported they stocked at least one of the next three most commonly mentioned brands. In addition, over 99% of pharmacies indicated they had emergency contraceptive pills in stock on the day of the audit, and just under 99% said they had condoms, while 83% of pharmacies said they had the injectable contraceptive Depo Provera.

Nearly three-quarters (72%) of pharmacies said they had a private space available to offer counseling in the pharmacy; however, 76% did not have any counseling materials available to assist pharmacy staff in counseling. Although available in only 10% of pharmacies, the most common materials were manuals and guides, with items such as flipcharts and counseling cards, available in fewer than 5% of pharmacies. Almost all pharmacies reported that OCPs (94%) and injectables (96%) required a prescription for purchase (see Table 3).

**Table 3.** Pharmacy characteristics (n=225).

Audit question	%
Private space available for counseling	72
Offering counseling on a specific method of family planning	54
At least one type of counseling material available (flipchart, poster, etc.)	24
Prescription required for oral contraceptive pills	94
Prescription required for injectable contraceptives	96

### Survey with personnel

In total, 49% of pharmacists and 47% of assistant pharmacists reported receiving training on family planning during their professional studies. A third of pharmacists and 12% of assistants reported being trained after their professional studies (the questions were not asked of counter staff); of these, 53% and 50%, respectively, had received training on counseling for family planning. Among those with any training on family planning counseling, all had received training on counseling for use of OCPs and 82% on injectables. Nearly 90% of all pharmacists and assistant pharmacists, including those who had received training on family planning previously, expressed a desire for training on counseling for family planning, and 86% in how to offer methods.

Table 4 outlines a series of questions asked to determine pharmacy staff knowledge of counseling issues related to OCPs. Scores ranged from 0–7 out of a possible 8 with an average of 1.6. Three staff (all pharmacists) (0.6%) were able to answer five or more questions correctly; 10% scored three points or more, and 44% scored two points or more. The average number of points earned on the OCP knowledge questions was slightly higher for those who reported receiving training of family planning during their professional studies (2.06) than those who did not report having received training (1.72).

**Table 4.** Staff knowledge questions, OCPs (n=486).

Questions and responses	% (n)	
**In general, at what time during the menstrual cycle do you recommend that the client begin using oral contraceptives?**		
Anytime as long as the client is reasonably sure she is not pregnant.	3 (13)	1 point
At the beginning of her cycle	78 (381)	
All other responses (anytime during her menstrual cycle, don’t know/no response)	19 (92)	
**In general, after finishing a 21-day pack of pills, how many days should the client wait before starting the next pack of pills?**		
7 days/one week	66 (322)	1 point
Other (Most common responses under “other: included “it depends,” and “start immediately.”)	16 (77)	
All other numerical responses	18 (87)	
**In general, after finishing a 28-day pack of pills, how many days should the client wait before starting the next pack of pills?**		
No wait/start immediately.	62 (302)	1 point
Other (The most common response under “other: was “I don’t know.”)	15 (73)	
All other numerical responses	23 (111)	
**In general, how many weeks after giving birth should a breastfeeding woman wait before starting combined oral** **contraceptives?**		
24 weeks/6 months	7 (32)	0.5 point
I don’t know	40 (192)	
All other numerical responses	54 (262)	
**In general, how many weeks after giving birth should a breastfeeding woman wait before starting progestin-only oral** **contraceptives?**		
Six weeks or earlier	18 (86)	0.5 point
I don’t know	49 (237)	
All other numerical responses	33 (163)	
**What are the most common side effects of oral contraceptives? ^a^**		
Five or more correct side effects	5 (25)	1 point
Four side effects listed	12 (57)	
Three side effects listed	24 (118)	
Two side effects listed	31 (151)	
One side effect listed	20 (99)	
Zero side effects listed	7 (36)	
**Which drugs or type of drugs reduce the effectiveness of oral contraceptive pills? ^b^**		
Two or more correct drugs listed	6 (29)	1 point
One correct drug listed	15 (74)	
Zero correct drugs listed	79 (383)	
**What are the reasons not to use oral contraceptive pills? ^c^**		
Five or more correct reasons listed	3 (12)	1 point
Four correct reasons listed	4 (17)	
Three correct reasons listed	13 (61)	
Two correct reasons listed	21 (104)	
One correct reason listed	26 (126)	
Zero correct reasons listed	34 (166)	
**In what situations should you recommend a back-up method for oral contraceptives? ^d^**		
Two or more correct situations listed	5 (23)	1 point
One situation listed	45 (219)	
Zero situations listed	50 (244)	

OCP, oral contraceptive pill ^a^ Correct responses include for headache, nausea, vomiting, vertigo, breast tenderness, spotting/breakthrough bleeding, weight gain. ^b^ Correct responses include seizure medicine, antiretroviral (ART), tuberculosis medicine ^c^ Correct responses include if client is pregnant, history of heart/circulation problems, breast cancer, severe liver disease, heavy smoker over 35 years old, breastfeeding first six months post-partum for combined OCPs, breastfeeding in first six weeks post-partum for Progestin-only OCPs, severe/complicated diabetes. ^d^ Correct responses include if client forgets 3 or more pills, if she vomited soon after taking pill, if she starts the pill after the first 5 days of her menstrual cycle, if she forgets to take Progestin-only pill by more than 3 hours

The majority (78%) of respondents’ knowledge adhered to the outdated protocol that a woman should initiate OCPs on the first day of her period, rather than current recommended practice, which allows women to start at any time during her cycle as long as she is reasonably sure she is not pregnant. While 59% of pharmacists and assistant pharmacists were able to name one or two common side effects, few (5.2%) were able to name five or more. While only 7% of respondents knew that a breastfeeding woman should wait twenty-four weeks/six months before initiating combined oral contraceptives, 18% knew that she could initiate progesterone-only pills either right away (current guidance), or after six weeks (previous guidance).

Table 5 outlines the series of questions asked to determine pharmacy staff knowledge of counseling issues related to injectable contraception. Scores ranged from 0–4 out of a possible 4 with an average of 0.9. Six staff (four pharmacists, one assistant pharmacist, and one counter staff) (1.2%), answered three or more questions correctly; 16% scored two or more points. As with OCPs, the average score was slightly higher for staff who reported having received training on family planning while in school. Those who reported receiving training averaged 1.1 points, versus 0.99 for those not reporting having received training.

**Table 5.** Staff knowledge questions, injectable contraceptives (n=484).

Questions and responses	% (n)	
**In general, at what time during the menstrual cycle do you recommend that the client begin using** **Depo Provera?**		
Anytime as long as the client is reasonably sure she is not pregnant.	5 (25)	1 point
No response/I don’t know	48 (235)	
All other responses (anytime during her menstrual cycle, don’t know/no response)	46 (224)	
**For how long is Depo Provera effective?**		
Three months/13 weeks	73 (305)	1 point
No response/I don’t know	19 (93)	
All other numerical responses	18 (86)	
**When should a woman return for a re-injection of Depo?**		
From 2 weeks before up to 4 weeks after	1 (4)	1 point
Up to 2 weeks before	1.4 (7)	0.5 point
Up to 4 weeks after	1 (4)	0.5 point
On the exact date	64 (313)	
All other responses	32 (153)	
**What are common side effects of Depo Provera? ^a^**		
Four or more correct side effects listed	10 (48)	1 point
Three correct side effects listed	21 (101)	
Two correct side effects listed	28% (137)	
One correct side effect listed	20 (99)	
Zero correct side effects listed	20 (99)	
**What are the reasons not to use Depo Provera? ^b^**		
Four or more correct reasons listed	4 (19)	1 point
Three correct reasons listed	7 (35)	
Two correct reasons listed	20 (98)	
One correct reason listed	25 (119)	
Zero correct reasons listed	44 (214)	

^a^ Correct responses include prolonged/heavy/irregular bleeding, amenorrhea, headaches, mood changes, weight gain, dizziness, abdominal pain/boating, reduced sex drive. ^b^ Correct responses include if client is pregnant, breast cancer, very high blood pressure, severe liver disease, unexplained vaginal bleeding, breastfeeding in first 6 weeks postpartum, thrombosis/blood clots, rheumatic lupus disease.

While nearly three-quarters (73%) of staff knew the duration of effectiveness to be three months per injection, few understood that women have a window from two weeks before to four weeks after her previous injection was set to expire to receive her reinjection. Whereas 21% of respondents could name three side effects associated with injectables, only 10% could name four. A total of 20% of respondents could name two reasons not to use injectables, but only 4% could name four or more reasons.

### Survey with clients

In total 21% of the entire sample (3,567) had ever used the pharmacy to get a contraceptive. Of clients currently using a modern family planning method, 31% used OCPs, 30% used injectables, 20% used implants and 10% used IUDs. A total of 70% of users obtained their methods through a public health facility, 19% from a private sector pharmacy and 10% from another source. Current users who procured their method through a private sector pharmacy received OCP (69%), condoms (21%), emergency contraceptive pills (5%) and injectables (3%).

Among the 390 current users who obtained a method from a pharmacy, 54 (14%) reported that they or their partner had received counseling at the pharmacy the last time they procured their method. Table 6 provides further information about that experience, including privacy and details of the counseling received. When asked, 52% of clients who had ever procured family planning through a pharmacy reported receiving a method without a prescription (390 of 741); 50% reported having bought OCPs and 2% injectables.

More clients reported having visual and auditory privacy for the counseling than what we found in our own audit—83% versus 72%. A majority reported having been treated with respect, and having had the opportunity to ask questions, whereas just over half reported that the staff explained how to use the method, asked them about contraindications to the method, and/or discussed side effects. Only 44% reported that the provided had talked to them about both advantages and disadvantages of the method.

**Table 6.** Clients’ most recent counseling experience received at pharmacy (n=54).

Survey question	% (n)
Counseling conducted with auditory and visual privacy	83 (45)
Staff treated client/partner with respect	87 (47)
Staff explained how to use the method	52 (28)
Staff asked about health conditions that might impede use of contraception	52 (28)
Staff spoke to client/partner about side effects	52 (28)
Staff discussed both advantages and disadvantages of the method	44 (24)
Staff gave client/partner the opportunity to ask questions	89 (48)

**Figure 1.**
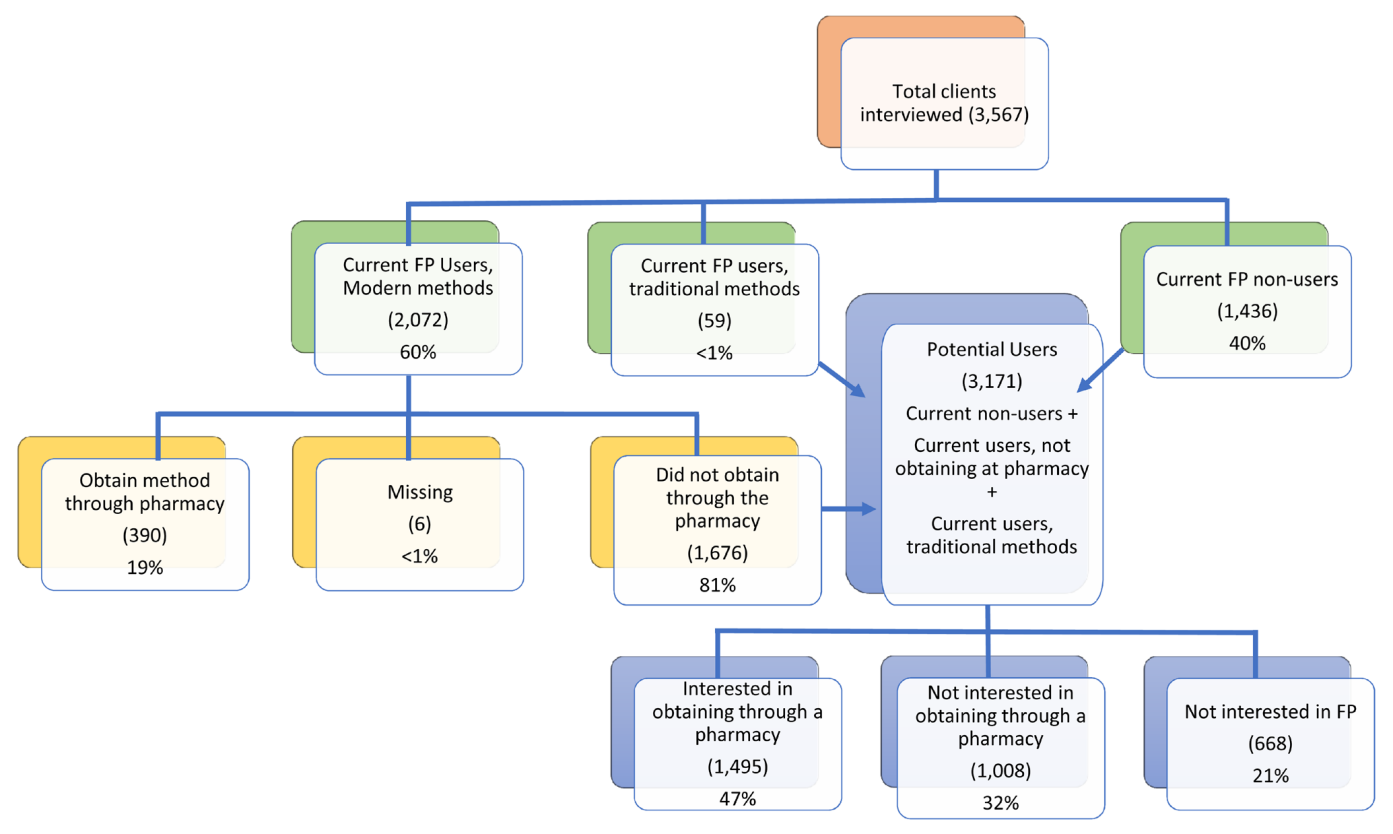
Client survey results flowchart.

Of potential users of the pharmacy for family planning, including current users procuring from a non-pharmacy source, users of traditional methods, and non-users of family planning who were interested in family planning (n=3,171), 47% said they would be interested in procuring a method through a pharmacy. Of those who were not interested in procuring through a pharmacy (n=1,008), 45% cited that they were dissatisfied with the quality of services through the pharmacy, and 12% reported high costs for their lack of interest. ( Figure 1)

**Figure 2.**
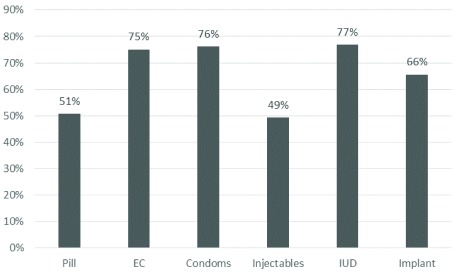
Percent of clients unwilling to pay for the given method at a pharmacy (n=1495).

Of the potential users of family planning who were interested in receiving a method through the pharmacy (n=1,495), half or more would not be willing to pay for their method in the pharmacy ( Figure 2). A total of 62% *would*, however, be willing to pay for counseling from pharmacy staff. The mean amount they would be willing to pay for counseling was 1679 CFA (approximately 3 USD) with a median and mode of 1000 CFA (approximately 2 USD).

## Discussion

Findings on the current state of family planning service provision through private sector pharmacies in the UHRI project area are mixed, especially in light of information on prior programs offering training to pharmacies. Both OCPs and injectables were in good supply, and close to three-quarters of pharmacies had a private space where counseling could be performed. Results on client-provider interactions are promising, as clients who procured their method through pharmacies reported being treated with respect and having the opportunity to ask questions.

At the same time, while a little over half of pharmacies indicated providing counseling in the pharmacy audit, only 14% of clients who procured their method through the pharmacy confirmed being counseled at their last visit, and few pharmacies had access to counseling materials such as posters, flip charts, or brochures. The limited family planning training provided to pharmacists and assistant pharmacists was reflected in some knowledge gaps, particularly with regards to up-to-date and detailed information. Clients who were counseled were not systematically informed of how to use their method, advantages and disadvantages or possible side effects. More detailed training on family planning counseling as part of the pharmacy licensure training program as well as opportunities for pharmacists and assistant pharmacists to refresh their skills through more broadly offered training could help address these challenges, especially given that providers in this study expressed interest in more training on methods and counseling. This expressed interest may be enough to overcome the “know-do” gap seen in other health fields ^25^. Others have also suggested creating linkages with and accountability to other FP providers to address the know-do gap ^26^.

Pharmacy staff in our study were aware of the law requiring a prescription to purchase OCPs and injectables, but our results suggested that it was not always adhered to, especially for OCPs but more rarely for injectables. In addition to women already procuring their method through pharmacies, we found there was latent demand for accessing family planning through pharmacies. Although the study design does not support a demand forecast, there was substantial interest among women in our sample (about half of future potential users of pharmacy for family planning) to supply through pharmacies. While some women currently procuring through pharmacies were able to obtain their method despite the prescription requirement, most were OCP users and very few obtained injectables. Given that injectables are the most widely used method in Senegal, revising the prescription requirement would be important to consider in the context of informed method choice, along with strengthening of pharmacists’ competencies, to fulfill the potential of urban private sector pharmacies in meeting this latent demand.

Price also warrants attention as it appears to be an important consideration for women. Price structure across service delivery channels in Senegal could also be a challenge on the supply side given the for-profit nature of pharmacy-based service provision. The majority of women interviewed were unwilling to pay for their method, though most *were* willing to pay for counseling obtained through the pharmacy, which reflects the typical payment structure of public health facilities, where women pay a small fee for methods (approximately a tenth of what they pay at private pharmacies), and a separate fee for a family planning consultation (less than a dollar at public facilities). Women in our sample expressed a willingness to pay 2–3 USD for the counseling at a pharmacy. To reconcile affordability with incentivization of private sector providers, additional interventions may need to be considered in Senegal to expand the potential of private sector pharmacies to reach women of all income levels, including increasing access to insurance and implementing savings products and voucher systems. A systematic review of family planning voucher programs in low and middle income countries concluded that in terms of contraceptive use, most programs reported a significant increase with vouchers ^27^.

Our study had several limitations. The ten UHRI districts represent urban settings, and findings may not transfer to other contexts. Because the sampling of urban private sector pharmacy clients was capped for non-users of family planning, we oversampled current users of family planning who obtain services in the pharmacy setting. While our finding indicate there is latent demand for services through pharmacies, results should not be interpreted as a precise estimate. We did not collect income information and can therefore not draw conclusions on pharmacies’ ability to improve access among the urban poor. Social desirability bias may have led to an inflated proportion of staff who reported requiring prescriptions. Other design options such as directly asking pharmacy staff about their practices or conducting a mystery client study were considered at the design stage, but met with opposition from ethics committees. Nevertheless, we attempted to address this shortcoming by triangulating pharmacy staff responses of knowledge of prescription requirements with client acknowledgement of having previously purchased a method without a prescription, creating a proxy for sales without a prescription. The scoring system for the knowledge index is crude and the passing score was based on the reasonable but subjective expectation that staff should be able to correctly answer half of the questions. Results on the proportion of knowledgeable staff should be interpreted cautiously, as this criterion may have been too stringent; however, this exercise allowed us to highlight important gaps in knowledge. Administering similar questions to facility-based staff could provide a useful comparison point in future research.

## Conclusion

Given high rates of success with task-sharing in other environments, the availability of contraceptive commodities and private counseling space within most urban-Senegalese pharmacies, positive client-pharmacist interactions, and client interest in receiving family planning directly through pharmacies, Senegal’s private sector urban pharmacies provide an opportunity to expand family planning service provision in urban areas. Proper training, including the use of job aids, is important to address current deficiencies in the quantity and quality of services provided. Although women reported being able to circumvent prescription requirements, they may still impose barriers to women seeking services, especially in terms of the ability of women to get their preferred method. Many medical experts consider such requirements unnecessary, and in countries in Asia, Europe, Africa and South and Central America, OCPs are available without a doctor’s prescription.

## Data availability

Replication data from this study are available on Harvard Dataverse: https://doi.org/10.7910/DVN/UQ3NWN ^21^

Data are available under the terms of the Creative Commons Zero "No rights reserved" data waiver (CC0 1.0 Public domain dedication).
