# Supplementary material for: Over the counter: The potential for easing pharmacy provision of family planning in urban Senegal
Source: Gates Open Res. 2019 May 15;2:29. Originally published 2018 Jun 13. [Version 3] doi: 10.12688/gatesopenres.12825.3 (PMC6600082; doi:10.12688/gatesopenres.12825.3)
Supplement: Peer review history

# Peer review: Reviewer response for version 3

**DOI:** 10.21956/gatesopenres.14137.r27362

**Published:** 2019-06-27

**Authors:** David K. Guilkey

**Affiliations:** 1 Department of Economics, Carolina Population Center, University of North Carolina at Chapel Hill, Chapel Hill, NC, USA

This paper reports descriptive results from a facility audit at a sample of 225 pharmacies in urban areas of Senegal. In addition the authors did exit interviews with a large number of clients of those facilities. The goal was to determine the potential for expanding access to family planning services in urban Senegal through pharmacies.

 The paper is well-written and the statistical methods are appropriate. I have a few comments that may improve the paper:

1. In 2011 and 2015, the Measurement, Learning, and Evaluation Project (also funded by the Gates Foundation) conducted surveys in a census to pharmacies as opposed to a sample in many of the same areas as this work. It may be useful to see how comparable the results of the facility audits are across the different surveys. See Cronin *et al.* (2018 ^1^) and https://tciurbanhealth.org/wp-content/uploads/2017/10/senegal_note_technique_mle_etudefinal2016_0.pdf [French].
2. It is not clear whether or not methods in stock were based on an interview with staff at the pharmacy or whether or not the methods were actually seen in stock by the interviewer. There is sometimes a discrepancy between these two assessments.
3. A total of 3,567 clients were interviewed. What was the refusal rate and is there any information on the characteristics of the women who refused to be interviewed?
4. There were a few typos in paper that should be cleared up before the final draft.

I confirm that I have read this submission and believe that I have an appropriate level of expertise to confirm that it is of an acceptable scientific standard.

## References

[1] : The effects of health facility access and quality on family planning decisions in urban Senegal. *Health Econ*.27(3) : 10.1002/hec.3615 576-591 10.1002/hec.3615 29094775PMC5867202

# Peer review: Reviewer response for version 2

**DOI:** 10.21956/gatesopenres.13970.r26809

**Published:** 2019-01-22

**Authors:** Katherine Tumlinson

**Affiliations:** 1 Department of Maternal and Child Health, Gillings School of Global Public Health, University of North Carolina at Chapel Hill, Chapel Hill, NC, USA; 2 Carolina Population Center, University of North Carolina at Chapel Hill, Chapel Hill, NC, USA

**Overall**: In my first review of this article, I noted that this is an interesting study that identifies some deficiencies in the quality and quantity of counseling provided to FP pharmacy clients while also suggesting some latent demand for procurement of FP through pharmacies, based on data collected at pharmacies in select urban areas of Senegal. I suggested several areas for improvement prior to publication in my first review and many of these were addressed. However, the following initial suggestions did not receive a response or need further clarification from the authors prior to acceptance for publication.

**Methods**:

1. **Initial comment**: The selection of districts could be presented with greater clarity. Did the URHI include only 10 districts for the country of Senegal and were all of the URHI districts in or near Dakar? If so, please make this clear. If not, how were the 10 districts in this analysis selected? Were the data for this study from the URHI endline survey in Senegal or were they part of a project piggybacking onto the Senegal URHI? **Comment following revision:** My interpretation of the revised version is that the data come from three cities (Dakar, Mbour, and Kaolack); could the authors just clarify why the other three URHI cities were excluded? Did these other cities not collect pharmacy-level data? Given data come entirely from urban areas, it would be helpful to insert the word “urban” in front of “Senegal” in the title.  Could you also clarify when the data were collected (2014?)?

**Results**

1. **Initial comment:** The first two sentences of the results section are difficult to interpret. Please clarify: how many facilities were initially selected, how many facilities refused to participate, and how many facilities were found to be closed (and were they permanently closed?). As written, it appears that 236 facilities were initially selected (236-18-18+25 = 225), yet this is different from the number (250) presented in the methods section. **Comment following revision: **Still unclear how many pharmacies were temporarily closed or not found.
2. **Initial comment: **Tables 4 and 5 list a number of survey questions that were used to create a score for provider knowledge of OCP and injectable provision. Please describe the logic and strategy behind the construction of this point system. For example, why should knowledge of five or more side effects (table 4) receive a point and all other categories receive no points? Is knowledge of five or more side effects meaningfully different than knowledge of four? How did the authors decide where to draw cut points? Has this approach been used or validated in prior studies? As these data comprise more than half of the total results section, it is important to understand the reason and logic behind this approach. **Comment following revision:** I was unable to find where the authors addressed this in the article.

**Discussion**

1. **Initial comment:** I would not agree that 72% is a “vast majority.” More than one in four pharmacies lacked a private space for counseling. I suggest just calling this a majority. **Comment after revision**: Not addressed.
2. **Initial comment:** In the first full paragraph on the left-hand side of page 9, the authors argue for increasing access to insurance, savings products, and voucher systems to help address price barrier in pharmacies. How feasible and realistic is this recommendation and would it serve to increase use of private facilities rather than pharmacies and drug-shops? **Comment following revision:** I was unable to find where the authors addressed this in the article.
3. **Initial comment:** The next sentence recommends addressing price sensitivities so that products are both profitable and affordable. Again, this begs the question, how feasible or realistic is this recommendation? To better understand the feasibility, it would be helpful to have information on the price currently paid by pharmacy versus facility clients as well as an understanding of what is meant by “price sensitivities should be addressed.” **Comment following revision:** I was unable to find where the authors addressed this in the article.
4. **Initial comment:** I recommend the authors include results of any similar studies in other countries or regions to place their findings in the context of existing knowledge. I also suggest presenting results of any other studies that have implemented the recommendations made by the authors such as programs that aim to decrease financial barriers – have these been successful in similar settings? This might help us to determine the degree to which these recommendations are realistic and/or likely to result in increased access to FP. **Comment following revision:** I was confused by the authors’ statement that their “results are difficult to fully put into context” without more information… does this mean there are no prior studies with any similarities to this study? Further, they don’t address the comment about whether their suggested interventions (insurance, savings products, voucher schemes) have had success in Senegal or other LICs.

**Conclusion**

1. **Initial comment: **The final concluding paragraph states that training and job aides are all that are needed to allow pharmacist to offer OCPs and injectables. Again, I would be careful not to overstate the results. The data, as presented, don’t tell us whether increased training and job aids would translate into an increased percentage of providers who offer counseling. Several recent studies suggest large gaps between provider knowledge/training and actual practice and there is evidence of job aids not being widely distributed and used.  I would encourage the authors to focus on the primary findings evident from the data:
  1. Nearly half of pharmacies aren’t offering method-specific counseling
  2. Providers also have low knowledge of correct provision of OCPs and injectables (assuming some of the scales are not overly stringent)
  3. Many clients are not being told how to use their method, about health conditions and side effects, or the advantages/disadvantages (Table 6!)
  4. The prescription requirement is a burden for some but more than half could procure a method without it
  5. A large percent of women appear to have latent demand for accessing FP through pharmacies and we assume they are not doing so due to the prescription requirement – yet more than half circumvented this requirement
    1. Price and quality appear to be a deterrent for many women; how have programs in similar contexts worked to address price and quality concerns in the pharmacy setting?

**Comment following revision: **Regarding the decision about whether or not to emphasize that half of women could circumvent the prescription requirement, I’m happy to agree to disagree ~ although I would encourage the authors to acknowledge this. However, more importantly, the conclusion continues to contain claims that aren’t supported by these data or by other studies and there is no acknowledgement of the growing literature that indicates provider trainings are not enough (see Mohanan’s recent article on the Know-Do Gap). ***In sum, the authors are recommending that Senegal lift the prescription requirement without providing evidence-based suggestions for how to address highly prevalent and valid price and quality concerns.***

 Also, please clarify, in the following statement in your author response sheet: “Given that half had, and we do not see resulting consequences on women’s health,…” were data on health outcomes for women receiving their method a the pharmacy analyzed and presented?

I confirm that I have read this submission and believe that I have an appropriate level of expertise to confirm that it is of an acceptable scientific standard, however I have significant reservations, as outlined above.

# Response

**Published:** 2019-05-07

**Authors:** Jill Peterson

Overall:

Thank you again for your careful review. Direct responses to your comments are here below, including clarification of where you can find our responses in the article.

Methods:

1. URHI included only 10 districts – the same as those in the present study. Under the section “Design and selection procedures,” we clarified that “We obtained a list of all private sector pharmacies (551) in ALL **10** URHI project districts.” Yes, we can add urban to the title. As stated under the *Data Collection* section, “Trained female data collectors conducted pharmacy audits, interviews with pharmacy staff, and client interviews between March and June 2015.”

Results:

1. We have revised the language in this section for clarification and specificity. 
2. We added details regarding the approach and acknowledging its limitations (see both methods and limitations). All questions were assumed to be of equal importance and given equal weight, and the passing score is based on the reasonable, but subjective expectation that staff would be able to correctly respond to half of the questions. Results on the proportion of knowledgeable staff should be interpreted with caution keeping this in mind, but the exercise was nonetheless useful in exposing knowledge gaps.

Discussion:

1. This was rephrased to “close to three-quarters.”
2. This paragraph has been edited and information about vouchers added. 
3. The price difference between public and private facilities was added as a parenthetical in the same paragraph ( *approximately a tenth of what they pay at private pharmacies*.) We have also added information on the cost of consultations at public facilities. It is a fine balance between making commodities affordable and competitive with the public facility rate and making it worth the effort for private pharmacies to stock them. It was those price sensitivities we suggested should be further studied when we added language on whether direct access to family planning through private sector pharmacies would bring in additional sales and profits to pharmacy owners. While on one hand the price may need to be less than the cost to the pharmacy of purchasing and stocking the items, the extra foot traffic may raise sales overall across the store. These are issues we were not able to explore within the time and budget of this study, but we agree that these issues should be further explored.   
4. The comment about context was actually in reference to the subjective nature of the cutoff for the scoring system. This was rephrased. We have added some information on vouchers and re-written the paragraph. 

 

Conclusion:

1. We have rephrased the discussion to be more nuanced. With regard to the prescription requirement, most women who were able to circumvent it were using COCs and few injectables, even though this is the most commonly used method in Senegal. We believe this may be important to consider in terms of method choice.

# Peer review: Reviewer response for version 1

**DOI:** 10.21956/gatesopenres.13897.r26518

**Published:** 2018-07-17

**Authors:** Barbara O'Hanlon

**Affiliations:** 1 O’Hanlon Health Consulting, LLC, NW Washington, DC, USA

I would also like the thank researches for the study. As a health policy expert working on integrating non-state provision of health services and systems with state (e.g. public) ones, I appreciate the growing research on private health sector potential contribution to key public health areas such as reproductive health and family planning. I believe this article on the Senegal experience in private pharmacy’s potential role will be a good addition to this growing body of literature on the private health sector in developing countries.

 Although my focus is primarily on harnessing and/or growing the private health sector to address public health objectives, it is difficult to truly understand a private sector role without understanding what is also happening at the same time in the public sector. Indeed, recent studies on consumer behavior indicate that most women do not go exclusively to one sector or the other but instead, “shop around for a better deal”. For example, women often go to private providers for ANC visits because of convenience and price but deliver their babies in a public facility because it is free. Similarly, AIDS patients often are tested, diagnosed and initiated on ARVs in the public sector but switch to the private sector for long-term care.

 Consequently, my main comment is I would like to know more about what is happening in the public sector before concluding that easing pharmacy provision will be an important strategy to address unmet need. Here are some areas to further pursue and/or clarify

- “Always, always, always” qualify what type (e.g. public vs. private and better yet if you can state public, not-for-profit and for-profit) of health facility as well as health cadres. When trying to understand if the private – or public sector as a matter of fact – is the strategic provider one needs to specify “who can do what”. For example, you state that pharmacists are currently forbidden from dispensing. Does this mean ***all*** pharmacists? Public pharmacists? Or only private pharmacists? In practice we see that regulations on task shifting are liberal for public health cadres than for similar cadres in the private sector. Therefore, it is critical to always specific. Similarly, you stated that according to regulations that a woman “is obliged to visit a health facility to initiate either of these PF methods”. Once again, can it be both types of facilities? Or just public? In South Africa only public doctors can diagnose and initiate AIDS. ***So it is important to specify because it has implications on a woman’s journey to receive FP methods.***
- You state prescriptions are required for OCPs but in practice women can obtain OCPs without a prescription at a private pharmacy. But few do: according to your data, 70% of women interviewed obtained their method in a public health facility (good you specified what type of facility!). ***So, what is going on here?*** Stock-outs in public facilities is the main reason women go directly to a private pharmacy but recent studies in Senegal demonstrate that stockouts in FP have almost disappeared due to IPM. Are women deciding to get their method in public facilities since that is where they get their prescription? Or is the price (consultation, method and opportunity cost) less than it would be in a private pharmacy? I would go back to the consumer data, if possible to understand why such an important number get their FP methods MOH facilities.
- Also, I would pursue further why non-users state they would go to a private pharmacy. Do they value convenience? Steady supply? Brand method? Cost?  Also, it is hard to see if cost (notice not price) is a factor unless there is some data on the consumer’s true cost to access FP in both public and private facility: some studies conclude that cost in the public sector (consultation fee and low price + transport cost and opportunity costs) can be equal if not greater than the **price **of OCP in a private pharmacy. So yes women may consider price too high but we do not know the true cost for them to get this same method in public sector.

 

 Without a better understanding of the interface between public and private supply as well as consumer preference, your conclusion that “Senegal pharmacies are well-positioned to meet the country’s increasing desire for modern contraception”. First, urban market – which the study only focused on – is very different than rural ones. Moreover, there are few private providers in rural and remote areas compared to urban and peri-urban ones. Second, the study did not explore private sector perspective on expanding FP methods are part of their product portfolio. I find your proposed “strategies” harness private pharmacies a bit simplistic. From a public health perspective, supplying them IEC materials as well as offering training to improve their counseling skills will help. But without more data on private pharmacy incentives (e.g. more foot traffic, spin-off sales in ancillary treatment areas like. Child health, higher margin on brand FP methods, etc.) you may not be able to “attract” sufficient number of private pharmacies to take the time (and money) to invest in improving counseling skills to offer clients among a crowded product portfolio. Research has demonstrated that in addition to training, other incentives are needed to attract private pharmacies: look at FP voucher programs where they receive donated FP products, can charge a “fair” dispensing fee to recuperate the cost of stocking the FP method, and in some cases, free marketing and community promotion).

**Is the work clearly and accurately presented and does it cite the current literature?**

 Partly. The author needs to go back to other studies on private pharmacy success stories to better understand under which context (e.g. policy, public supply, consumer preference, financing) a private pharmacy can address unmet need. Also, the author should look at the incentives to attract private pharmacies to deliver FP methods.

**Is the study design appropriate and is the work technically sound?**

 Yes, but it would be great if they could go back to the consumer data to get more information on consumer preference between public and private supply. Also, it would have been optimal if the research had also included some questions (directed to pharmacy owner / manager) to better understand their market conditions to get at the issue of incentives.

**Are sufficient details of the methods and analysis provided to allow replication by others?**

 Partly, but I would have expanded the questions to include more questions on consumer preferences, private provider perspective. I would also add a summary of the public supply to understand how the private supply is different and therefore could be a complimentary strategy to the public one to increase access to FP methods. Finally, if the budget permitted I would add some elements of cost and price comparison between the two sectors.

**If applicable, is the statistical analysis and its interpretation appropriate?**

 Yes, for the data they collected. I would like to see if they could “squeeze” a bit more on consumer preference, cost/price and private provider perspective.

**Are the conclusions drawn adequality supported by the results?**

 Partly. I think the conclusions are a bit over stated and recommendations a bit too simplistic.

I confirm that I have read this submission and believe that I have an appropriate level of expertise to confirm that it is of an acceptable scientific standard, however I have significant reservations, as outlined above.

# Response

**Published:** 2018-12-07

**Authors:** Jill Peterson

Thank you for your thoughtful and excellent comments.  We have addressed nearly all of them. In a few cases we were unable to make changes because of data limitations. 

 

1. We have clarified terminology to “private-sector pharmacies,” as appropriate.
2. Given prescription requirements at the time of our study, women would have had to go to a facility to get a prescription for a method, and methods are provided for low or no cost through public facility pharmacies.  While we did not conduct interviews at public facility pharmacies, as it was outside the scope of our research, we can assume these are main reasons women obtain family planning through public facilities.  As discussed in our results, women who do not seek family planning at the pharmacy cited quality concerns as well as cost concerns.  Future research could include more questions about why women go through the various options.
3. We agree that more research could be done on incentives for private sector pharmacies to provide low-cost family planning methods. We regret not including questions on private provider perspectives and consumer preferences.
4. We have clarified language in the conclusion with regards to the influence of pharmacies in the urban setting, as opposed to nationwide.

# Peer review: Reviewer response for version 1

**DOI:** 10.21956/gatesopenres.13897.r26519

**Published:** 2018-07-10

**Authors:** Katherine Tumlinson

**Affiliations:** 1 Department of Maternal and Child Health, Gillings School of Global Public Health, University of North Carolina at Chapel Hill, Chapel Hill, NC, USA; 2 Carolina Population Center, University of North Carolina at Chapel Hill, Chapel Hill, NC, USA

**Overall**: This is an interesting study presenting descriptive results of pharmacy audits and interviews with pharmacy staff and exiting pharmacy clients in Dakar, Senegal. Results of this study indicate there is room for improvement in terms of (some aspects of) the quality and quantity of counseling provided to FP pharmacy clients and that there is latent demand among women in Dakar for procurement of FP through pharmacies. The authors suggest this latent demand could be fulfilled by addressing concerns about price, quality, and by removing laws that restrict OTC provision. However, results also suggest that laws requiring a prescription before obtaining a contraceptive method from a pharmacy are not universally enforced – more than half of participants were able to procure FP without a prescription. As such, it’s difficult to determine from the information provided which barriers are most significant and critical to address. Additionally, the authors do not clearly describe how they define and measure the quality of services and, in several places, it is difficult to determine the number or percent of women falling into key categories. Further, the discussion section includes statements that contradict or overstate the information provided in the results section. Additional minor suggestions are described below. I encourage the authors to revise their manuscript in response to the feedback below and thank them for their contribution.

**Introduction**:

1. Please briefly define or describe “drug shops” and how they differ from pharmacies.
2. Pharmacies are described as “plentiful” in urban/peri-urban areas, in contrast to health facilities; it would be helpful to have further description or statistical information on the prevalence/accessibility of pharmacies compared to health facilities.
3. The sentence that begins (paragraph three), “Moreover, when compared with the alternative of pregnancy…” doesn’t fit well with the rest of the paragraph which describes the benefit of pharmacies offering OCPs and injectables. I would recommend pulling out this sentence and creating a separate paragraph which transparently describes and addresses some of the possible downsides or concerns regarding OTC provision of FP through pharmacies.
4. Per my comment above, please provide at a short paragraph that describes some of the possible downsides or existing concerns regarding drug shop/pharmacy OTC provision of FP.

**Methods**:

1. The selection of districts could be presented with greater clarity. Did the URHI include only 10 districts for the country of Senegal and were all of the URHI districts in or near Dakar? If so, please make this clear. If not, how were the 10 districts in this analysis selected? Were the data for this study from the URHI endline survey in Senegal or were they part of a project piggybacking onto the Senegal URHI?
  - Assuming all included districts/pharmacies are in or near Dakar, please discuss how this impacts generalizability of results in the limitations section.
2. It is not clear how specific pharmacies within each district were selected for inclusion. For example, within each district, were the pharmacies selected at random? Please describe.
3. Under the pharmacy staff paragraph, line 5, please delete the word “not” in front of present. I believe the sentence should read: “If none of the three were present the day of the interviews, that position was not included.”
4. One of the key objectives of the study (as stated in the abstract and in the middle of the right-hand column of page 3) is to assess the quality of the services provided through pharmacies; yet the authors do not provide a definition of quality or describe how quality is measured in this study. This should be included. There are many different ways to define and measure quality and many possible aspects of quality that could be included. The authors need to unpack how and why they chose the specific indicators presented in the descriptive analysis (and how and why other indicators are excluded from this analysis) and tie this justification to an existing framework such Bruce, Donabedian, or other framework.

**Results**

1. The first two sentences of the results section are difficult to interpret. Please clarify: how many facilities were initially selected, how many facilities refused to participate, and how many facilities were found to be closed (and were they permanently closed?). As written, it appears that 236 facilities were initially selected (236-18-18+25 = 225), yet this is different from the number (250) presented in the methods section.
2. Tables 4 and 5 list a number of survey questions that were used to create a score for provider knowledge of OCP and injectable provision. Please describe the logic and strategy behind the construction of this point system. For example, why should knowledge of five or more side effects (table 4) receive a point and all other categories receive no points? Is knowledge of five or more side effects meaningfully different than knowledge of four? How did the authors decide where to draw cut points? Has this approach been used or validated in prior studies? As these data comprise more than half of the total results section, it is important to understand the reason and logic behind this approach.
3. Is the total sample 3,567 (first sentence of section entitled ‘Survey with clients’) or 3,569 as listed in abstract and title of Table 2?
4. Page 7, right column, second paragraph begins: “Among the 390 current users who obtained a method from a pharmacy…” However, in the abstract we’re told that 60% of the sample of 3,569 women use FP and 11% *of these* obtained their method from a pharmacy: (3,569 x .6) x .11 = @235 women, not 390. Please explain why these two numbers are different. If the total of number of women who are current users who obtained a method from a pharmacy is 11% of the total sample, please correct the abstract by removing the phrase “of these.”
5. The last two paragraphs are difficult to interpret as the various numbers presented don’t sum to the expected result. Please provide a flow chart or graphic to visually display which participants were contraceptive users versus non-users and the latent demand among non-users. As currently written, it’s difficult to understand (for example, see above point regarding 235 vs 390). In trying to understand the latent demand, I read that there are 3,074 women who are non-users or users who did not get their method from a pharmacy (which would imply there are 493 women who are current users who did get their method from a pharmacy?). It states that 47% of these 3,074 are interested in procuring through a pharmacy. This would cause me to conclude that 53% of these 3,074 are not interested in procuring through a pharmacy. Yet, 53% of 3,074 is 1,629 and the authors tell us that the actual number is 1,008. Please review all numbers, make corrections as appropriate, and provide a visual (or more detailed description) to help the readers understand the behavior and preferences of the sample of women.
6. Regarding the presentation of results on quality of services, Tables 4 and 5 relate primarily to the provider’s technical competence which is one aspect of quality, but there are many other components of quality for which the authors appear to have data but don’t describe or discuss. For example, Table 6 presents results that tie to additional aspects of quality such as respectful treatment and provision of information; yet Table 6 is barely mentioned in the results section – just one sentence directing readers to look at Table 6. Quality is also barely mentioned in the discussion section. Notably, 34% of those not interested in procuring FP services through a pharmacy cited poor quality as the reason – and presumably they are not talking about components of technical competence such as those listed in tables 4 and 5 since that is difficult for a client to assess. Hence, I would suggest the data presented in Table 6 deserve more unpacking.

**Discussion**

1. The first sentence states that pharmacies largely adhere to the law requiring a prescription to purchase OCPs and injectables yet the results of the client interviews indicate that more than half of clients who have ever obtained a method from a pharmacy did not have a prescription. How do the authors explain this contradiction between the first sentence of the discussion section and the actual results of their study? As transparently acknowledged in the limitations section, it is possible that the pharmacy staff may have been reluctant to report they were breaking the law by not requiring a prescription and therefore the data from the exiting clients may be more accurate than the data from providers on this point.
2. I would not agree that 72% is a “vast majority.” More than one in four pharmacies lacked a private space for counseling. I suggest just calling this a majority.
3. Pharmacies are described as an “important point of supply” although just 11 percent of current users report getting their method from a pharmacy and only 22% report ever using pharmacies for FP supply. Some may agree that these percentages warrant calling pharmacies an important point of supply and others may disagree, but I would urge the authors to be careful not to overstate the results of their study.
4. For the phrase “including new or re-starting users” – I don’t recall seeing this nuance presented in the results. If this nuance is important, please present these data.
5. In the first full paragraph on the left-hand side of page 9, the authors argue for increasing access to insurance, savings products, and voucher systems to help address price barrier in pharmacies. How feasible and realistic is this recommendation and would it serve to increase use of private facilities rather than pharmacies and drug-shops?
6. The next sentence recommends addressing price sensitivities so that products are both profitable and affordable. Again, this begs the question, how feasible or realistic is this recommendation? To better understand the feasibility, it would be helpful to have information on the price currently paid by pharmacy versus facility clients as well as an understanding of what is meant by “price sensitivities should be addressed.”
7. I recommend the authors include results of any similar studies in other countries or regions to place their findings in the context of existing knowledge. I also suggest presenting results of any other studies that have implemented the recommendations made by the authors such as programs that aim to decrease financial barriers – have these been successful in similar settings? This might help us to determine the degree to which these recommendations are realistic and/or likely to result in increased access to FP.
8. Please mention the limited geographic scope (just Dakar, correct?) as a potential limitation.

**Conclusion**

1. The authors conclude that pharmacies are well-positioned to meet demand for FP in Senegal, but my understanding is that this study was conducted only in Dakar. Please clarify the abstract and methods section if this understanding is incorrect. Otherwise clarify in the conclusion that the generalizable population is Dakar and not Senegal: I believe more than 80% of the country’s population live outside of Dakar.
2. The final concluding paragraph states that training and job aides are all that are needed to allow pharmacist to offer OCPs and injectables. Again, I would be careful not to overstate the results. The data, as presented, don’t tell us whether increased training and job aids would translate into an increased percentage of providers who offer counseling. Several recent studies suggest large gaps between provider knowledge/training and actual practice and there is evidence of job aids not being widely distributed and used. I would encourage the authors to focus on the primary findings evident from the data:
  - Nearly half of pharmacies aren’t offering method-specific counseling
  - Providers also have low knowledge of correct provision of OCPs and injectables (assuming some of the scales are not overly stringent)
  - Many clients are not being told how to use their method, about health conditions and side effects, or the advantages/disadvantages (Table 6!)
  - The prescription requirement is a burden for some but more than half could procure a method without it
  - A large percent of women appear to have latent demand for accessing FP through pharmacies and we assume they are not doing so due to the prescription requirement – yet more than half circumvented this requirement
    - Price and quality appear to be a deterrent for many women; how have programs in similar contexts worked to address price and quality concerns in the pharmacy setting?

I confirm that I have read this submission and believe that I have an appropriate level of expertise to confirm that it is of an acceptable scientific standard, however I have significant reservations, as outlined above.

# Response

**Published:** 2018-12-07

**Authors:** Jill Peterson

Thank you also for your review and your valuable comments.  We have addressed nearly all comments in the revised draft.  We have noted the changes below and made a few further comments of our own.  

Introduction: All comments addressed.

Methods:

1. District information clarified.  This study was not part of the URHI endline survey; it was conducted independently.
2. Language around selection clarified.
3. Language clarified.
4. Language added to better describe quality.

 

Results:

1. Language clarified and corrected.
2. Logic and strategy behind provider scoring added.
3. The total sample should be 3,567.  
4. “Of these” deleted.
5. Flow chart added and language clarified within the text.
6. More information on quality and specifically, on Table 6, added.

Discussion:

 We agree that this was confusing to read.  We clarified in the first sentence that most providers are “aware of” prescription requirements.
Suggestion taken.
Language edited so as not to overstate.
Language edited.
6) and 7)  The questions around price barriers are good ones.  Our suggestion is that incentives such as vouchers be used in private sector pharmacies.  We did add language around current prices in private sector pharmacies versus public facilities, and clarified where we see a need for further research in this area.  Unfortunately, relevant research in Senegal or similar countries is limited, but we hope it will be explored in the future. 

1. Language clarified to note that the research was done in 8 districts of Dakar as well as two urban areas outside of Dakar.

Conclusion:

1. The research was done in 8 districts of Dakar as well as two urban areas outside of Dakar.
2. We have edited the language in our conclusion based on your suggestions, but we stand by the overall conclusion that the prescription requirement be lifted.  The glass is nearly half full and conversely, half empty.  While approximately half of women could circumvent the prescription requirement, half had not.  Given that half had, and we do not see resulting consequences on women’s health, we believe private sector pharmacies can provide this service, and in fact many already are. As such, private sector pharmacies should be allowed to do so without fear of punishment for breaking the law.
